# Phase Equilibrium Relations in the Sc_2_O_3_-Ga_2_O_3_ System

**DOI:** 10.6028/jres.067A.003

**Published:** 1963-02-01

**Authors:** S. J. Schneider, J. L. Waring

## Abstract

The phase equilibrium diagram was determined for the Sc_2_O_3_-Ga_2_O_3_ system. A quenching furnace, wound with 60 percent Pt—40 percent Rh wire, was employed for experiments conducted at temperatures up to 1,800 °C. An induction furnace, having an iridium crucible susceptor, was used to obtain higher temperatures. Temperatures in the quenching furnace were measured with both an optical pyrometer and a 95 percent Pt—5 percent Rh versus 80 percent Pt—20 percent Rh thermocouple. The melting point of Ga_2_O_3_ was determined as 1,795 ±15 °C. Experiments at temperatures as high as 2,405 °C failed to melt Sc_2_O_3_. Two intermediate binary phases, a compound believed to be 6Sc_2_O_3_·5Ga_2_O_3_ and a solid solution occur in the system. The solid solution phase appears as a single phase in the region roughly defined by the compositional limits of 55 to 73 mole percent Ga_2_O_3_ at the solidus. The 6:5 compound, stable only at high temperatures, melts incongruently at 1,770 ±15 °C and decomposes below 1,700 ±15 °C. The compound appears to have orthorhombic symmetry with *a*=13.85 A, *b*= 9.80 A, and *c*=9.58 A. The indicated uncertainties in the melting points are a conservative estimate of the overall inaccuracies.

## 1. Introduction

A recent publication by Schneider, Roth, and Waring [1]^1^ outlined the general subsolidus phase relations for binary combinations of oxides of the trivalent cations. The paper was intended to provide a basis for subsequent detailed phase equilibrium studies of systems which might be representative of a particular group of systems. As part of a series of binary systems selected for study from those delineated in the previous work, the present investigation reports the phase equilibrium relations in the Sc_2_O_3_-Ga_2_O_3_ system.

Scandium sesquioxide (Sc_2_O_3_) is cubic with *a*=9.845 A [2] and has the C-type rare earth oxide structure (Tl_2_O_3_). It has no known polymorphs. The melting point of Sc_2_O_3_ has not been previously reported although it was estimated to be about 2,300 °C [3].

Several polymorphic forms of Ga_2_O_3_ are known to exist [4]. The only stable modification, commonly referred to as beta gallia, is monoclinic with *a*=12.23 A, *b*=3.04 A, *c*=5.80 A, and *β*=103.7° [5]. Goldschmidt and coworkers [6] estimated the melting point of Ga_2_O_3_ to be approximately 1,900 °C. Von Wartenberg and Reusch [7] reported a value of 1,740 °C. The latest melting point determination on Ga_2_O_3_ was that of Hill, Roy, and Osborn [8] who reported 1,725 ±15 °C.

## 2. Materials

The starting materials used in this study were found by general qualitative spectrochemical analysis 2 to have the following impurities:

Sc_2_O_3_—Y present in amounts less than 1.0 percent. La, Mn, Si, Yb, and Zr; each present in amounts less than 0.1 percent. Al, Ca, Cr, Cu, Fe, Mg, and Pb; each present in amounts less than 0.01 percent. Ag present in amounts less than 0.001 percent.
Ga_2_O_3_—Al and Si; each present in amounts less than 0.01 percent. Ca, Cu, Fe, Mg, Ni, Pb, and Sn; present in amounts less than 0.001 percent. Ag present in amounts less than 0.0001 percent.

## 3. Apparatus and Test Methods

A specially designed quenching furnace as well as an induction furnace were used in the present study. The quenching furnace is of interest because of its suitability for extended use at temperatures up to 1,800 °C.^3^ It consisted essentially of two concentric alumina tubes encased in a “Transite” framework and insulated with alumina grain. The inner tube (o.d.—½ in., 24 in. long) and the outer tube (o.d.—2 in., 17 in. long) were wound nine turns per inch with 20 gage, 60 percent Pt–40 percent Rh wire, and 20 gage, 80 percent Pt–20 percent Rh wire, respectively. Separate power sources were used with each of the two windings. The power to the outer winding (booster) was supplied from a variable transformer. No attempt was made to control the temperature of the booster other than with approximate settings of the transformer. An a-c bridge type controller [10] in which the furnace winding was one arm of the bridge was used to obtain and control the temperature of the inner winding. As indicated by the thermocouple, temperatures in the hot zone of the furnace were easily controlled to ±3 °C.

The specimen was placed in a small (o.d.—3 mm, i.d.—2.6 mm, ½ in. long) 80 percent Pt—20 percent Rh tube which in turn was suspended from the quenching hooks in the hot zone of the inner tube by 0.005 in. diam 60 percent Pt—40 percent Rh wire. The hooks were made from 20 gage 60 percent Pt—40 percent Rh wire. A 90 percent Pt—10 percent Rh versus 100 percent Pt thermocouple was used to measure temperatures below 1,650 °C. For measurements between 1,650 and 1,800 °C, both a 95 percent Pt—5 percent Rh versus 80 percent Pt—20 percent Rh thermocouple and an optical pyrometer were used. It is noteworthy that it was necessary to shield the thermocouple with 90 percent Pt—10 percent Rh foil in order to reduce the pickup of induced emf at high temperatures. Even when the shielding was adequately grounded, the use of electronic equipment to record the output of the thermocouple was impossible. To overcome this problem, a high precision potentiometer and spotlight galvanometer were employed to measure the d-c emf of the thermocouple.

The optical pyrometer was sighted through a calibrated 45° glass prism using the bottom of the quenching hook-thermocouple assembly as the target. The platinum alloy tube could not be seen and thus approximate blackbody conditions probably prevailed. In every instance, the thermocouple and pyrometer values agreed to within 10 °C.

The essential features and characteristics of the induction furnace used in this work has been previously described [11]. In essence, the induction furnace simply consisted of an iridium crucible and cover which acted as the susceptor. The crucible with cover had the following overall nominal dimensions: height*—*¾ in., o.d.— 716 in., and wall thickness*—* 364 in. A small fragment of a sintered pellet was placed on a small iridium button which in turn was set inside the crucible. Temperatures were controlled manually and were measured with an optical pyrometer sighted through a calibrated 45° glass prism and the 364 in. diam hole in the crucible cover. As verified by calibration data, blackbody conditions were apparently obtained.

The temperature measuring systems of both the quenching furnace and the induction furnace were frequently calibrated against the melting points of Au (1,063 °C), Pd (1,552 °C), and Pt (1,769 °C).^4^ In addition, the measuring system of the induction furnace was also calibrated against the melting point of Rh (1,960 °C). Temperatures measured in either furnace were considered to be accurate to at least ±5 °C below 1,650 °C and ±15° above. Precision of the measurements were better than ±5 °C.

Specimens were prepared from either 0.75 or 1 g batches of various combinations of Sc_2_O_3_ and Ga_2_O_3_. Calculated amounts of each oxide, corrected for ignition loss at 1,000 °C, were mechanically mixed, pressed at 10,000 lb/in.^2^ into a ⅜ in. diam pellet and fired on Pt foil at 1,350 °C a minimum of 6 hr. The specimens were then ground in an agate mortar, remixed, again pressed into pellets and fired at 1,600 °C for 12 hr. The pellets, without regrinding, were again fired at 1,650 °C a minimum of 12 hr and in some cases as long as 30 hr.

Subsolidus as well as some melting point data were obtained by the familiar quenching technique, Following the preliminary heat treatments, the specimens were ground, placed in the platinum alloy tubes and fired at various temperatures for different periods of time in the quenching furnace. The tube containing the specimen was quenched into ice water and then examined by X-ray diffraction at room temperature using a high-angle recording Geiger-counter diffractometer and Ni-filtered Cu radiation. Equilibrium was assumed to have been attained when the X-ray pattern showed no change after successive heat treatments of a specimen or when the data were consistent with the results from a previous set of experiments.

Solidus temperatures were established using both the platinum alloy quenching furnace and the iridium crucible induction furnace. Because of the temperature limitations of the quenching furnace, melting points above 1,800 °C were determined exclusively with the induction unit. However, below 1,800 °C, some duplicate determinations were performed in each of the furnaces.

Solidus temperatures were determined by visual observation of quenched or rapidly cooled specimens which had been held at a given temperature approximately 30 to 60 sec. Powdered samples in platinum alloy tubes were used with the quenching furnace determinations while small fragments of a sintered pellet set directly on the iridium button were used as test specimens in the induction furnace. Melting of the specimen was indicated by a combination of evidence. A partially or completely melted specimen characteristically slumped very slightly and invariably adhered to the side of the platinum alloy tube or iridium button. In addition, the specimen had a translucent, pearly to clear appearance. Solidus temperatures, as recorded, are estimated to be accurate to ±15 °C.

Liquidus temperatures were practically impossible to determine because of the high viscosity of the liquid phase. In essence, the physical appearance of a partially melted specimen did not differ appreciably from that of a completely melted sample. X-ray patterns of melted specimens were extremely diffuse and difficult to interpret. A consistent correlation between the degree of melting and the X-ray pattern of a specimen could not be made.

## 4. Results and Discussion

**Figure 1:**
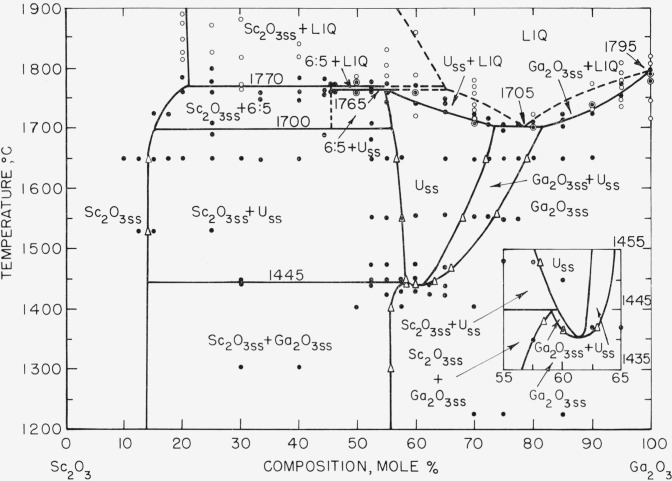
Phase equilibrium diagram for the *Sc_2_O_3_-Ga_2_O_3_* system Insert shows expanded view of diagram from 55 to 65 mole percent Ga_2_O_3_ and from 1,485 °C to 1,455 °C. U—Solid solution phase of unknown structure type. Liq.—Liquid. ss—Solid solution. ●—Compositions and temperatures of experiments conducted in the quench furnace. ○—Compositions and temperatures of experiments conducted in the iridium crucible induction furnace. △—Boundary limits as determined by the parametric method. ⊙—Compositions and temperatures of experiments conducted in both the quenching and induction furnaces.

The equilibrium phase diagram for a major portion of the Sc_2_O_3_-Ga_2_O_3_ system is given in figure 1. It was constructed from the data listed in tables 1 and 2. The solid circles represent the compositions and temperatures of experiments conducted in the quench furnace. Open circles indicates those studied in the induction unit. Triangles represent the boundary limits of solid solution areas as determined by the parametric method [12]. The liquidus curve given in the figure is considered to be a reasonable estimate of the true liquidus.

Because of the temperature limitations of the equipment, only the melting point of Ga_2_O_3_ could be established. The melting point of Sc_2_O_3_ has been estimated to be between the melting points of BeO and Al_2_O_3_, about 2,300 °C [3]. However, all attempts to melt Sc_2_O_3_ in the present work at temperatures as high as 2,405 °C proved unsuccessful. The melting point of Ga_2_O_3_ was determined to be 1,795 ±15 °C. This value compares rather unfavorably with the 1,725 ±15 °C value reported by Hill, Roy, and Osborn [8]. The determination of Hill et al. [8], was made on a strip furnace which is generally subjected to considerable random errors. To verify that the melting point of Ga_2_O_3_ was at least above 1,769 °C, the melting point platinum, a small piece of Pt ribbon was imbedded in pure Ga_2_O_3_ and heated in the quench furnace to 1,775 °C. Upon examination, the platinum ribbon was completely melted while the Ga_2_O_3_ showed no signs of melting.

Two intermediate binary phases occur in the Sc_2_O_3_-Ga_2_O_3_ system. The first, a compound believed to be 6Sc_2_O_3_-5Ga_2_O_3_, melts incongruently at 1,770 ±15 °C. The compound has a fairly narrow region of temperature stability, decomposing to two solid phases at 1,700 ±15 °C. The compound could only be formed in appreciable amounts after heating appropriate compositions for at least 10 hr at temperatures between 1,760 °C and its melting point. It was necessary to quench the specimens from these temperatures in order to retain the phase at room temperature. Slow or even rapid cooling resulted in decomposition of the phase. Even these long time heats, a few degrees below the melting point, failed to produce completely single phase specimens. Due to either the inability to obtain equilibrium or to the failure to completely “quench in” the compound, residual amounts of other phases persisted. Possible compositions of the compound considered most likely were the Sc_2_O_3_:Ga_2_O_3_ molar ratios of 5:4, 6:5, and 7:6 with the 6:5 ratio most probable. X-ray patterns of the three possible compositions indicated that the compound formed in the greatest amounts relative to the minor phases at the 6Sc_2_O_3_:5Ga_2_O_3_ composition. It should be emphasized that the line in the phase diagram (fig. 1) representing the compound is dashed not because of uncertainty in the existence of the compound but because of uncertainty in its exact composition.

A literature search did not reveal any other compound or solid solution in binary combinations of trivalent oxides which were isostructural with the 6:5 compound. A comparison of the X-ray pattern of the 6:5 compound with that of Sc_2_O_3_ indicated that, because of similar positions and intensities of major reflections, a similarity in structure might exist. The X-ray pattern of the 6:5 compound, given in table 3, was indexed on an orthorhombic basis with *a*=13.85 A, *b*=9.80 A, and *c*=9.58 A. There is generally fair agreement between observed and calculated 1*/d*^2^ values. If the indexing given in table 3 represents the true indexing, the structure of the 6:5 compound apparently is an orthorhombic distortion of the cubic C-type structure where $a\left(6:5\right)≅\sqrt{2a}\left({\mathrm{Sc}}_{2}O_{3}\right)$.

The X-ray pattern of the second intermediate binary phase found in the system has been previously reported by Schneider, Roth, and Waring [1]. It is a solid solution and may possibly have a structure similar to kappa alumina, a metastable polymorph of Al_2_O_3_ [1]. The X-ray pattern of this solid solution phase could not be indexed, so that verification of its possible kappa alumina-like-structure could not be obtained. This phase (labeled U_ss_ in fig. 1) occurs in the Sc_2_O_3_–Ga_2_O_3_ system from about 55 to 73 mole percent Ga_2_O_3_ at the solidus. Solidus temperatures in this region ranged between approximately 1,764 and 1,705 °C. The compositional area of stability decreases at lower temperatures, finally pinching out with a minimum at about 61.5 mole percent Ga_2_O_3_ and 1,440 °C. An expanded view of the minimum is given in the inset of figure 1.

The remainder of the system is comprised of solid solutions of the end member oxides as well as appropriate two phase areas. Solid solution of Ga_2_O_3_ in Sc_2_O_3_ (C-type structure) occurs from 0 to about 21 mole percent Ga_2_O_3_ at the solidus. Solidus temperatures in this area extend from the melting point of Sc_2_O_3_ (unknown) to 1,770 °C, the incongruent melting point of the 6:5 compound. The amount of Sc_2_O_3_ solid solution decreases to about 15 mole percent Ga_2_O_3_ at 1,300 °C.

X-ray patterns of specimens having compositions and heat treatments generally corresponding to the Sc_2_O_3ss_+U_ss_ region (fig. 1) characteristically showed one, sometimes two diffraction peaks (*d*=2.7216 A, *d*=2.4212 A) which normally are not associated with those of the primary phases. The extraneous peaks were seemingly not related to experimental procedure; that is, longer heat treatment nor quenching or slow cooling of the specimens changed the intensities of the peaks. Furthermore, if the reflections represented a true equilibrium phase, they would follow the lever rule principle, increasing or decreasing with composition. This was not the case however. At present, the only possible explanation is that the extraneous reflections indicate the presence of some unidentified metastable phase.

Solid solution of Sc_2_O_3_ in Ga_2_O_3_ (beta-gallia structure type) occurs from about 82 to 100 mole percent Ga_2_O_3_ at the solidus. The solidus temperatures vary between 1,705 °C, the eutectic temperature, and 1,795 °C, the melting point of Ga_2_O_3_. Contrary to normal expectations, the amount of Ga_2_O_3_ solid solution increases at lower temperatures, extending to approximately 56 mole percent Ga_2_O_3_ at 1,405 °C. The unit cell dimensions of Ga_2_O_3_ solid solution at about 56 mole percent Ga_2_O_3_ and 1,405 °C were *a*=12.70 A, *b*=3.16 A, *c*=5.92 A and 0 = 102° 18′ in contrast to pure Ga_2_O_3_ where *a*=12.23 A, *b*=3.04, *c*=5.80 A, and *β*=103.9° [5]. This large change in the cell dimensions of pure Ga_2_O_3_ due to solid solution resulted in the separation of normally unresolved X-ray diffraction peaks. Insofar as can be determined, the unit cell dimensions for the Ga_2_O_3_ solid solution are the largest thus far reported for a phase having the beta gallia structure type.

Specimens which normally should have contained only two phases, U_ss_+Ga_2_O_3ss_, generally contained, upon slow cooling, an additional phase. This third phase was identified as Ga_2_O_3ss_, but having different unit cell dimensions than the true equilibrium Ga_2_O_3ss_ phase. This phenomena of differential exsolution was common for specimens high in Ga_2_O_3_ content which had been slow cooled in the preliminary calcinations. To obtain the true equilibrium phases it was necessary to rely on the results obtained only from quenched specimens.

The exact compositions of the peritectic and eutectic points which occur in the system could not be determined accurately because of the inability to obtain complete liquidus data.

## 5. Summary

The equilibrium phase diagram for a major portion of the Sc_2_O_3_-Ga_2_O_3_ system was determined from a study of solid state reactions amd melting point relations. A specially designed platinum alloy quenching furnace was employed for subsolidus and melting point experiments conducted below 1.800 °C. A 95 percent Pt-5 percent Rh versus 80 percent Pt-20 percent Rh thermocouple and an optical pyrometer were used to measure temperatures between 1,650 and 1,800 °C. An inductively heated iridium crucible was used for the determination of solidus and liquidus temperatures above 1,800 °C. Phases were identified by examination of X-ray diffraction patterns.

The melting point of Ga_2_O_3_ was found to be 1,795 ±15 °C. The indicated uncertainty in the melting point is a conservative estimate of the overall inaccuracy. The melting point of Sc_2_O_3_ could not be determined because of temperature limitations of the equipment. However, it does not melt below 2,405 °C, the highest temperature attained in the present study.

Two intermediate binary phases were formed in the system. The first, a compound of approximately 6:5 molar ratio, melts incongruently at 1,770 °C and decomposes below 1,700 °C. Its X-ray pattern was indexed on the basis of an orthorhombic cell with *a*=13.85 A, *b*=9.80 A, *c*=9.58 A. This cell appears to be related to that of Sc_2_O_3_ with *a* (6:5) $≅\sqrt{2}a\;\left({\mathrm{Sc}}_{2}O_{3}\right)$. The second binary phase, a solid solution, exists from about 55 to 73 mole percent Ga_2_O_3_ at the solidus. The compositional range of stability decreases at lower temperatures, pinching out with a minimum at 61 mole percent Ga_2_O_3_ and 1,440 °C. The X-ray pattern of the solid solution phase could not be indexed.

## Figures and Tables

**Table 1:** Experimental quenching data for compositions in the *Sc*_2_*O*_3_*—Ga*_2_*O*_3_ system

Composition		Heat treatment^a^		X-ray diffraction analyses^b^	Remarks
Sc_2_O_3_	Ga_2_O_3_	Temp.	Time		
					
*Mole*%	*Mole*%	*°C*	*hr*		
90	10	1650	2	Sc_2_O_3ss_	
87.5	12.5	1530	6.5	Sc_2_O_3ss_	
		1650	2	SC_2_O_3ss_	
85	15	1530	6.5	Sc_2_O_3ss_^c^	
		1650	2	SC_2_O_3ss_^d^	U_ss_ not detected.
		1727	4.5	SC_2_O_3ss_	
82.5	17.5	1650	2	SC_2_O_3ss_+U_ss_^c^	Similar results obtained on specimen furnace cooled from 1,650 °C.
		1727	4.5	Sc_2_O_3ss_+6:5	
80	20	1650	2	SC_2_O_3ss_+U_ss_^d^	Similar results obtained on specimen furnace cooled from 1,650 °C.
75	25	1530	6.5	SC_2_O_3_88+U_ss_^c^	
		1650	2	Sc_2_O_3ss_+U_ss_^c^	Similar results obtained on specimen furnace cooled from 1,650 °C.
		1692	4.5	SC_2_O_3ss_+U_ss_^c^	
		1709	3.5	Sc_2_O_3ss_+U_ss_+6:5^c^	Reheat of 1,650 °C specimen nonequilibrium; 6:5 forming with U_ss_ decreasing in amount relative to previous heat.
		1760	4	Sc_2_O_3ss_+6:5	
70	30	1304	64	SC_2_O_3ss_+Ga_2_O_3_ss	
		1441	24	SC_2_O_3ss_+U_ss_	Nonequilibrium.
		1441	68	Sc_2_O_3ss_+Ga_2_O_3ss_	Nonequilibrium; U_ss_ decreasing in amount relative to previous heat.
				+U_ss_	
		1447	68	Sc_2_O_3ss_+U_ss_^d^	
		1447	68	Sc_2_O_3ss_+U_ss_+Ga_2_O_3ss_	Nonequilibrium; reheat of 1,304 °C specimen.
		1650	2	SC_2_O_3ss_+U_ss_^d^	
66.667	33.333	1747	3.5	Sc_2_O_3ss_+6:5+U_ss_	Nonequilibrium.
		1757	4.5	Sc_2_O_3ss_+6:5+U_ss_	Reheat of 1,747 °C specimen; nonequilibrium; U_ss_ decreasing in amount relative to previous heat.
		1642	5	SC_2_O_3ss_+U_ss_^c^	Reheat of 1,757 °C specimen.
60	40	1304	64	Sc_2_O_3ss_+Ga_2_O_3ss_	
		1650	2	U_ss_+Sc_2_O_3ss_^d^	
		1747	4	6:5+Sc_2_O_3ss_+U_ss_	Reheat of 1,650 °C specimen; nonequilibrium.
		1760	4	6:5+Sc_2_O_3ss_	Reheat of 1,747 °C specimen.
		1768	1	6:5+Sc_2_O_3ss_+U_ss_	Nonequilibrium.
55. 56	44.44	1758	4	6:5+U_ss_+Sc_2_O_3ss_	Calcined only at 1,300 °C for 20 hr; nonequilibrium.
(5:4)					
		1763	9	6:5+U_ss_+Sc_2_O_3ss_	Calcined only at 1,300 °C for 20 hr; nonequilibrium; U_ss_ and Sc_2_O_3ss_ barely detectable.
		1689	3	U_ss_+Sc_2_O_3ss_^d^	Calcined only at 1,300 °C for 20 hr; specimen heated first to 1,761 °C for 1 hr then cooled to 1,689 °C.
54.545	45.455	1761	10.5	6:5	Calcined only at 1,350 °C for 16 hr; may be small amount of U_ss_ present.
(6:5)					
53.846	46.154	1760	5	6:5+U_ss_+Sc_2_O_3ss_	Calcined only at 1,350 °C for 20 hr; nonequilibrium.
(7:6)					
		1762	4.5	6:5+U_ss_	Reheat of 1,760 °C specimen.
50	50	1405	16	Ga_2_O_3ss_4-Sc_2_O_3ss_	Calcined only at 1,350 °C for 20 hr.
		1758	4	6:5+U_ss_	Calcined only at 1,350 °C for 20 hr.
47.5	52. 5	1443	6	Ga_2_O_3ss_+Sc_2_O_3ss_+U_ss_	Nonequilibrium; U_ss_ present in very small amounts.
		1447	68	U_ss_+Sc_2_O_3ss_	
		1471	21	U_ss_+Sc_2_O_3ss_^d^	
		1555	2	U_ss_+Sc_2_O_3ss_^c^	
47.5	52.5	1650	2	U_ss_+Sc_2_O_3ss_^d^	Similar results obtained on specimen furnace cooled from 1,650 °C.
		1760	5	U_ss+_6:5	
		1648	4	U_ss_+Sc_2_O_3ss_^d^	Reheat of 1,760 °C specimen.
		1680	4.5	U_ss_+Sc_2_O_3ss_^d^	Reheat of 1,760 °C specimen.
		1709	4	U_ss_+6:5	Reheat of 1,760 °C specimen.
45	55	1423	66	Ga_2_O_3ss_+Sc_2_O_3ss_	
		1450	19	U_ss_+Sc_2_O_3ss_	
		1471	21	U_ss_+Sc_2_O_3ss_^d^	
		1555	2	U_ss_+Sc_2_O_3ss_^d^	
		1650	2	U_ss_+Sc_2_O_3ss_	
		1742	2	U_ss_	
42.5	57.5	1405	16	Ga_2_O_3ss_	
		1429	18	Ga_2_O_3__ss_	
		1440	19	Ga_2_O_3ss_+U_ss_	Nonequilibrium.
		1453	19	U_ss_+Sc_2_O_3ss_	
		1559	2	U_ss_	
		1650	2	U_ss_	
40	60	1429	18	Ga_2_O_3ss_	
		1442	220	Ga_2_O_3ss_+U_ss_	
		1450	19	U_ss_	
		1475	4	U_ss_	
		1559	2	U_ss_	
37.5	62.5	1423	156	Ga_2_O_3ss_	
		1442	220	Ga_2_O_3ss_+U_ss_	
35	65	1423	156	Ga_2_O_3__ss_	
		1442	220	Ga_2_ O_3__ss_	
		1471	21	Ga_2_O_3ss_+U_ss_	
		1559	2	U_ss_	
		1650	2	U_ss_	
30	70	1227	94	Ga_3_O_3ss_	
		1402	2	Ga_2_O_3ss_	
30	70	1555	2	U_ss_+Ga_2_O_3ss_	
		1650	2	U_ss_	
27.5	72.5	1555	2	Ga_2_O_3ss_+U_ss_	
		1650	25	U_ss_+Ga_2_O_3ss_	
		1650	30	Ga_2_O_3ss_(I)+Ga_2_03_ss_(II)+U_ss_	Specimen not quenched; nonequilibrium. The Ga_2_O_3ss_ phases have different unit cell dimensions.
25	75	1227	94	Ga_2_O_3ss_	
		1550	2	Ga_2_O_3__ss_	
		1650	2	Ga_2_O_3ss_+U_ss_	
		1650	24	Ga_2_O_3ss_(I)+Ga_2_O_3ss_(II)	Specimen not quenched; nonequilibrium. The Ga_2_O_3_ phases have different unit cell dimensions.
22.5	77.5	1550	2	Ga_2_O_3ss_	Reheat of 1,650 °C specimen.
		1650	2	Ga_2_O_3ss_+U_ss_	
		1550	30	Ga_2_O_3_8_ss_(I)+Ga_2_O_3ss_(II)	Specimen not quenched; nonequilibrium. The Ga_2_O_3ss_ phases have different unit cell dimensions.
20	80	1650	2	Ga_2_O_3ss_	Same results obtained on specimen furnace cooled from 1,650 °C.
15	85	1227	94	Ga_2_O_3ss_	
		1650	2	Ga_2_O_3__ss_	Same results obtained on specimen furnace cooled from 1,650 °C.
10	90	1650	2	Ga_2_O_3ss_	Same results obtained on specimen furnace cooled from 1,650 °C.

a Unless otherwise indicated, all specimens calcined at 1,350 °C for 6 hr, 1,600 °C for 12 hr and at 1,650 °C for a minimum of 12 hr prior to quenching experiment.

b Phases identified are given in order of amount present at room temperature: ss—solid solution; U—solid solution of unknown structure type; 6:5—compound having approximate composition of 6 Sc_2_O_3_:5Ga_2_O_3_.

c Extraneous X-ray peaks at *d*=2.7216 A and *d*=2.4212 A; probably represent nonequilibrium phase.

d Extraneous X-ray peak at *d*=2.7216 A; probably represents nonequilibrium phase.

**Table 2:** Melting characteristics of the Sc_2_O_3_—Ga_2_O_3_ system

Composition^a^		Temperature^b^	Furnace^c^	Observation^d^
SC_2_O_3_	Ga_2_O_3_			
				
*Mole* %	*Mole* %	°*C*		
100	0	2345	I	Not melted.
		2405	I	Do.
90	10	1975	I	Do.
		2010	I	Do.
80	20	e 1760	Q	Do.
		1784	Q	Do.
		1814	I	Do.
		1828	I	Do.
		1849	I	Do.
		1874	I	Do.
		1890	I	Do.
		1970	I	Do.
		2030	I	Partially melted.
		2073	I	Do.
75	25	1760	Q	Not melted.
		1778	Q	Partially melted.
		e 1800	Q	Do.
		1825	I	Do.
		1870	I	Do.
70	30	e 1763	I	Not melted.
		1771	I	Partially melted.
		1819	I	Do.
		1882	I	Do.
60	40	1768	Q	Not melted.
		1786	Q	Partially melted.
		1818	I	Do.
		1842	I	Do.
55.56	44.44	e 1761	Q	Not melted.
(5:4)		e 1763	Q	Do.
		1782	Q	Partially melted.
54.545	45.455	e 1761	Q	Not melted.
(6:5)		1771	Q	Partially melted.
53.846	46.154	e 1762	Q	Not melted.
(7:6)		1774	Q	Partially melted.
50	50	1757	I	Not melted.
		e 1758	Q	Do.
		1775	Q	Partially melted.
		1778	I	Do.
		1788	I	Do.
47.5	52.5	e 1760	Q	Not melted.
		1767	Q	Partially melted.
		1777	Q	Do.
		1778	Q	Do.
45	55	e 1760	Q	Not melted.
		1775	Q	Partially melted.
		1789	I	Do.
		1805	I	Do.
		1831	I	Do.
		1982	I	Completely melted?
40	60	1722	I	Not melted.
		1742	Q	Do.
		1758	Q	Partially melted.
		1760	I	Partially melted.
40	50	1771	Q	Do.
		1790	I	Do.
		1861	I	Completely melted?
35	65	1728	Q	Not melted.
		1741	Q	Partially melted.
		1748	Q	Do.
30	70	1708	Q	Not melted.
		1710	I	Do.
		1714	I	Partially melted.
		1727	Q	Do.
		1731	I	Do.
		1738	I	Do.
		1758	I	Completely melted?
		1768	I	Do.
		1779	I	Do.
27.5	72.5	1709	Q	Not melted.
		1715	Q	Partially melted.
25	75	1697	Q	Not melted.
		1707	Q	Partially melted.
22.5	77.5	1704	Q	Not melted.
20	80	1701	Q	Do.
		1703	I	Do.
		1707	Q	Partially melted.
		1724	I	Completely melted?
15	85	1703	Q	Not melted.
		1715	Q	Partially melted.
		1725	Q	Do.
10	90	1725	Q	Not melted.
		1730	I	Do.
		1735	Q	Partially melted.
		1737	I	Do.
		1785	I	Completely melted?
5	95	1736	I	Not melted.
		1749	I	Do.
		1754	Q	Do.
		1769	I	Partially melted.
		1771	Q	Do.
		1782	I	Do.
		1794	I	Completely melted.?
		1810	I	Do.
0	100	1717	I	Not melted.
		1748	I	Do.
		1775	I	Do.
		e 1775	Q	Do.
		1789	I	Do.
		1789	Q	Do.
		1793	I	Do.
		1797	Q	Completely melted?
		1799	I	Do.
		1809	I	Do.
		1820	I	Do.

a Specimens calcined at 1,350 °C for 6 hr, 1,600 °C for 12 hr, and at 1,650 °C a minimum of 12 hr prior to melting point experiment.

b Specimens furnace cooled except when indicated.

c Q—quenching furnace; I—induction furnace.

d Definite verification of complete melting could not be obtained.

e Specimen quenched.

**Table 3:** *X-ray diffraction powder data for* 6Sc_2_O_3_: 5Ga_2_O_3_^a^ (CuK*α* radiation)

*hkl*^b^	*d*^c^	1/*d*^2^		*I*/*I*_0_^e^
		Obs	Cal^d^	
				
	*A*			
002	4.80	0.0434	0.0436	5
320	3.354	.0888	.0885	18
401	3.257	.0943	.0943	7
103	3.109	.1035	.1032	3
222	3.058	.1070	.1061	15
402	2.805	.1271	.1269	100
040	2. 4505	.1665	.1665	14
004	2.3956	.1742	.1742	23
403	2.3492	.1812	.1814	13
114	2.2956	.1898	.1899	3
233	2.1721	.2120	.2125	11
224	2.0535	.2371	.2367	4
404	1.9721	.2572	.2576	3
622	1.9132	.2732	.2728	2
350	1.8043	.3072	.3072	4
025	1.7859	.3135	.3139	4
252	1.7555	.3245	.3247	10
800	1. 7326	.3331	.3334	23
542	1.7175	.3390	.3403	11
722 044	}1.7134	.3406	$\left\{ \begin{array}{l} .3405\\ .3408 \end{array}\right.$	34
624	1. 5736	.4038	.4035	7
116	1. 5663	.4076	.4077	6
740 360 900	}1.5396	.4219	$\left\{ \begin{array}{l} .4218\\ .4217\\ .4220 \end{array}\right.$	4
803 910	} 1.5214	.4320	$\left\{ \begin{array}{l} .4315\\ .4324 \end{array}\right.$	3
605	1.4748	.4598	.4598	32
406	1.4507	.4752	.4754	20
804	1.4427	.5073	.5077	6
171	1.3789	.5260	.5262	3
017	1. 3547	.5449	.5440	6
027	1.3196	.5742	.5742	3
080	1.2251	.6663	.6663	7
282	1.1703	.7301	.7306	3
672	1.1618	.7408	.7402	4

a Specimen not single phase; reflections due to phases other than ≈6:5 compound are deleted.

b Orthorhombic Miller indices.

c Interplanar spacing.

d Based on orthorhombic cell, *a* = 13.85 A, *b*=9.80 A, *c*=9.58 A.

e Relative intensity.

## Footnotes

1 Figures in brackets indicate the literature references at the end of this paper.

2 The spectrochemical analyses were performed by the Spectrochemistry Section of the National Bureau of Standards.

3 All quoted temperatures referring to the present work are given on the International Practical Temperature Scale of 1948 (Text Revision 1960) [9].

4 The purity of the metals used for calibration purposes was better than 99.9 percent.
